# Combined therapy of transarterial chemoembolization and stereotactic body radiation therapy versus transarterial chemoembolization for ≤5cm hepatocellular carcinoma: Propensity score matching analysis

**DOI:** 10.1371/journal.pone.0206381

**Published:** 2018-10-31

**Authors:** Baek Gyu Jun, Sang Gyune Kim, Young Don Kim, Gab Jin Cheon, Koon Hee Han, Jeong-Ju Yoo, Young Seok Kim, Soung Won Jeong, Jae Young Jang, Sae Hwan Lee, Suyeon Park, Hong Soo Kim

**Affiliations:** 1 Department of Internal Medicine, University of Ulsan College of Medicine, Gangneung Asan Hospital, Gangneung, South Korea; 2 Department of Internal Medicine, Soonchunhyang University College of Medicine Bucheon Hospital, Bucheon, South Korea; 3 Department of Internal Medicine, Soonchunhyang University College of Medicine Seoul Hospital, Seoul, South Korea; 4 Department of Internal Medicine, Soonchunhyang University College of Medicine Cheonan Hospital, Cheonan, South Korea; 5 Department of Biostatistics, College of Medicine, Soonchunhyang University, Seoul, Korea; Texas A&M University, UNITED STATES

## Abstract

Patients with liver cirrhosis and hepatocellular carcinoma (HCC) are often ineligible for resection or local ablation therapy due to poor liver function and/or difficult location. The aim of this study is to evaluate therapeutic outcomes of stereotactic body radiotherapy (SBRT) combined with transarterial chemoembolization (TACE) compared with TACE alone for HCC measuring less than 5 cm. From March 2011 to December 2016, 85 patients underwent SBRT with TACE (SBRT-TACE group) and 114 underwent TACE (TACE group) at 4 tertiary hospitals. Local control rate (LCR), progression-free survival (PFS) and overall survival (OS) were compared after propensity-score matching (1:1 ratio). The SBRT-TACE group showed significantly higher 1- and 3-year LCR than the TACE group (91.1% and 89.9%, respectively vs 69.9% and 44.8%, respectively; *P* < 0.001). The SBRT-TACE group showed better 1- and 3-year PFS than the TACE group (56.5% and 32.3%, respectively vs 42.2% and 21.6%, respectively; *P* = 0.022). However, 1-, 3- and 5-year OS was not different between the SBRT-TACE and TACE groups (98.8%, 89.1% and 80.7%, respectively vs 99.7%, 83.3% and 71.0%, respectively; *P* = 0.206). In multivariate analysis, the overall SBRT added to TACE did not contribute to extend PFS. However, in patients with less than 2 tumors, the combined therapy was effective (HR 0.590, 95% CI 0.392–0.889, *P* = 0.012). SBRT-TACE is superior to TACE in terms of LCR. Particularly, SBRT-TACE may be an effective alternative in patients with HCC number (≤2), which is not indicated for resection or local ablation.

## Introduction

Hepatocellular carcinoma (HCC) is the third most common cause of cancer-related death worldwide [1]. Resection is the standard treatment for early-stage HCC [2]. However, many patients are not indicated for resection or ablative therapy because of advanced cirrhosis or tumor location [3]. Surgery is not indicated for elderly patients in poor general condition. Transarterial chemoembolization (TACE) is usually performed as an alternative treatment [4, 5]. The effect of TACE is well established especially in patients with Barcelona clinic liver cancer (BCLC) stage B. Unfortunately, the response rate of conventional TACE is relatively low (40%), and therefore, regarded as a palliative treatment [6, 7].

Traditionally, radiotherapy (RT) played a limited role due to radiation-induced liver disease (RILD) [8]. Recently, stereotactic body radiotherapy (SBRT) has emerged as a new modality of HCC treatment. Technological advances allow RT using high doses of radiation to conform to the target volume safely [9, 10]. Compared with conventional RT as a palliative approach, which is associated with low local control (LC), stereotactic body radiotherapy (SBRT) results in a high rate of LC, by delivering a high dose of radiation in a few fractions to small HCC [11]. Further, adjuvant SBRT following TACE is an effective treatment modality in relatively medium-sized HCC [12]. With advances in radiation technology, RILD after SBRT treatment was tolerable even in patients with Child-Pugh (CP) score ≤7 [13].

However, the efficacy of SBRT combined with TACE compared with TACE alone is unknown. In this study, we investigated the effect of SBRT and TACE combination versus TACE alone on tumor response and patient survival.

## Methods

### Patients

Data of HCC patients who underwent TACE as an initial treatment between March 2011 and February 2016 were reviewed at four tertiary referral hospitals (Soonchunhyang University Seoul, Bucheon, Cheonan Hospital, and Gangneung Asan Medical Center). Patients following the inclusion criteria were selected: 1) tumor size ≤5 cm of long diameter, and ≤3 lesions present; 2) ineligible for resection or local ablative therapies; and 3) CP class A or B. The exclusion criteria were as follows: 1) previous treatment of resection or radiofrequency ablation or TACE; 2) extrahepatic metastasis; and 3) presence of vascular invasion or portal vein tumor thrombosis. The diagnosis of HCC was made by using dynamic imaging technique [14].

One hundred fourteen patients were treated with TACE alone (TACE group) while eighty five patients were treated with TACE in combination with SBRT (TACE-SBRT group). The selection criteria for TACE and SBRT were mainly determined by considering tumor vascularity, hepatic angiography, accessibility, risk of bleeding or liver toxicity. We conducted propensity score matching to minimize the differences in underlying confounding factors between the two groups (1:1 ratio). This study was approved by the Institutional Review Board of Soonchunhyang University Seoul, Bucheon, Cheonan Hospital, and Gangneung Asan Medical Center, and written informed consent was waived because of the retrospective study.

### TACE

TACE was performed via the common femoral artery using an angiographic catheter followed by selection of feeder vessels of hepatic segments. Patients were treated with a mixture of intra-arterial adriamycin (50 mg/m^2^) and lipiodol (5 to 10 mL) with gelfoam embolization at Soonchunhyang University Seoul, Cheoan, Bucheon Hospital and Gangeung Asan Hospital [15, 16].

### SBRT

SBRT was carried out at Soonchunhyang University Seoul Hospital using the CyberKnife Radiosurgery System (Accuray Incorporated, Sunnyvale, CA), Soonchunhyang University Cheonan using the Novalis TX (Varian Medical Systems and BrainLab), Soonchunhyang University Bucheon using the TomoTherapy device (Madison, WI, USA) and Gangneung Asan Medical Center using the TrueBeam medical linear accelerator (Varian Medical Systems, Palo Alto, CA, USA). SBRT was performed viable tumors that showed incomplete response after first TACE based on follow-up CT. Patients were immobilized in supine position with arms above their head. Gross tumor volume (GTV) was measured based on CT images at the end-expiratory phase fused with multi-phase MR images. Extension based on movement within the gating phase (30–70%) from the GTV was set as the internal target volume (ITV). The planning target volume (PTV) was defined as the volume with a 5 mm margin added to the ITV. A total dose of 40–60 Gy (median, 55 Gy) was administered in the PTV of three to five fractions over consecutive days or twice a week [13].

### Liver toxicity

Liver toxicity was defined as worsening of CP score by 2 or more within 3 months or elevated liver transaminases more than five times the upper normal limit after treatment [17].

### Assessment

The primary endpoint included comparison of the overall survival (OS) in the SBRT-TACE and TACE groups. The secondary endpoint was the comparison of LC and progression-free survival (PFS).

All patients were followed up every 1 to 3 months. Physical examinations, complete blood cell counts, biochemical profiles, tumor markers, and three-phasic CT or magnetic resonance imaging (MRI) scans were performed at every follow-up visit. Complications were assessed according to version 4 of the Common Terminology Criteria for Adverse Events. Liver toxicity was defined as elevated liver transaminases more than five times the upper normal limit or worsening of CP score by 2 or more within 3 months after SBRT [17].

### Statistical analysis

The OS, PFS and LC in each treatment group were estimated using the Kaplan-Meier method and log-rank test. OS was calculated from the date of diagnosis until the date of final follow-up or death. PFS was estimated from the date of initial TACE until the date of extra- and/or intrahepatic disease progression, recurrence, or death. LC was defined as the absence of progressive disease (PD) within the PTV as per Response Evaluation Criteria in Solid Tumors (RECIST) v1.1 in multiphasic CT or MRI. Lesions that developed or progressed outside the PTV in the liver or lymph nodes were scored as regional PD and those developed in other organs as distant PD. Survival and control times were calculated from the start of SBRT. Time to progression and survival were evaluated with the Kaplan-Meier method [18]. Cox proportional-hazards model was used to evaluate the factors influencing PFS and OS rates.

To reduce the effect of potential confounding in a retrospective study, we also performed rigorous adjustment for differences in baseline characteristics of patients using propensity score methods (R version 3.1.2, ‘MatchIt’ package). The SBRT-TACE and TACE groups were matched 1:1 to maximize the propensity score match. Age, gender, tumor size, number of tumors, Child-Pugh score, and BCLC stage were selected on the basis of this score, and calculated from baseline characteristics. A P-value of < 0.05 was considered significant. All statistical analyses were performed using SPSS statistical package (version 18.0; SPSS Inc., Chicago, IL, USA).

## Results

### Patient characteristics before and after propensity score matching

After propensity score matching at a 1:1 ratio, the SBRT-TACE and TACE groups comprised 85 patients, respectively. No significant differences in sex (*P* = 0.858), age (*P* = 0.894), tumor number (*P* = 0.816), tumor size (*P* = 0.753), etiology (*P* = 0.778) and CP score (*P* = 0.663) were observed (Table 1).

**Table 1:** Baseline characteristics before and after propensity score matching.

	Before propensity matching			After propensity matching	
Variable	SBRT-TACE (n = 85)	TACE (n = 114)	P-value	TACE (n = 85)	P-value
Sex					
Male	65	88	0.905	64	0.858
Female	20	26		21	
Mean age (mean ± SD)	62.6 ± 10.0	63.32 ± 10.1	0.639	62.8 ± 10.6	0.894
Number			0.045		0.816
1	55	55		51	
2	20	33		23	
3	10	26		11	
Mean tumor size (mean ± SD)	2.23 ± 1.17	2.54 ±1.35	0.095	2.29 ± 1.17	0.753
Mean total tumor size	3.05 ± 1.79	3.58 ± 2.34	0.074	2.94 ± 2.01	0.691
Number of TACE	3.57 ± 2.64	3.10 ± 2.52	0.268	3.18 ± 2.54	0.328
Child-Pugh score (mean ± SD)	5.52 ± 0.85	5.57 ± 1.18	0.183	5.59 ± 1.06	0.633
Child-Pugh class A	71	96	0.897	74	0.516
B	14	18		11	
BCLC stage					
0	22	32	0.054	29	0.476
A	55	58		50	
B	8	24		6	
Etiology					
Alcohol	22	27	0.920	18	0.778
Hepatitis B virus	47	65		51	
Hepatitis C virus	11	13		9	
others	5	9		7	
ALT (mean ± SD)	27.7 ± 24.3	29.4 ± 18.8	0.674	29.1 ± 18.1	0.666
Total bilirubin (mg/dl) (mean ± SD)	0.94 ± 0.56	0.95 ± 0.65	0.857	0.90+0.63	0.654
Platelet count (x10^9/^L) (mean ± SD)	128 + 63.5	116 ± 53	0.160	118 ± 53	0.261
Prothrombin time (INR) (mean ± SD)	1.14 ± 0.20	1.17 ± 0.18	0.283	1.15 ± 0.19	0.651

ALT alanine transaminase, INR = International Normalized Ratio

### Local control, progression-free survival and overall survival after propensity score matching

**Fig 1:**
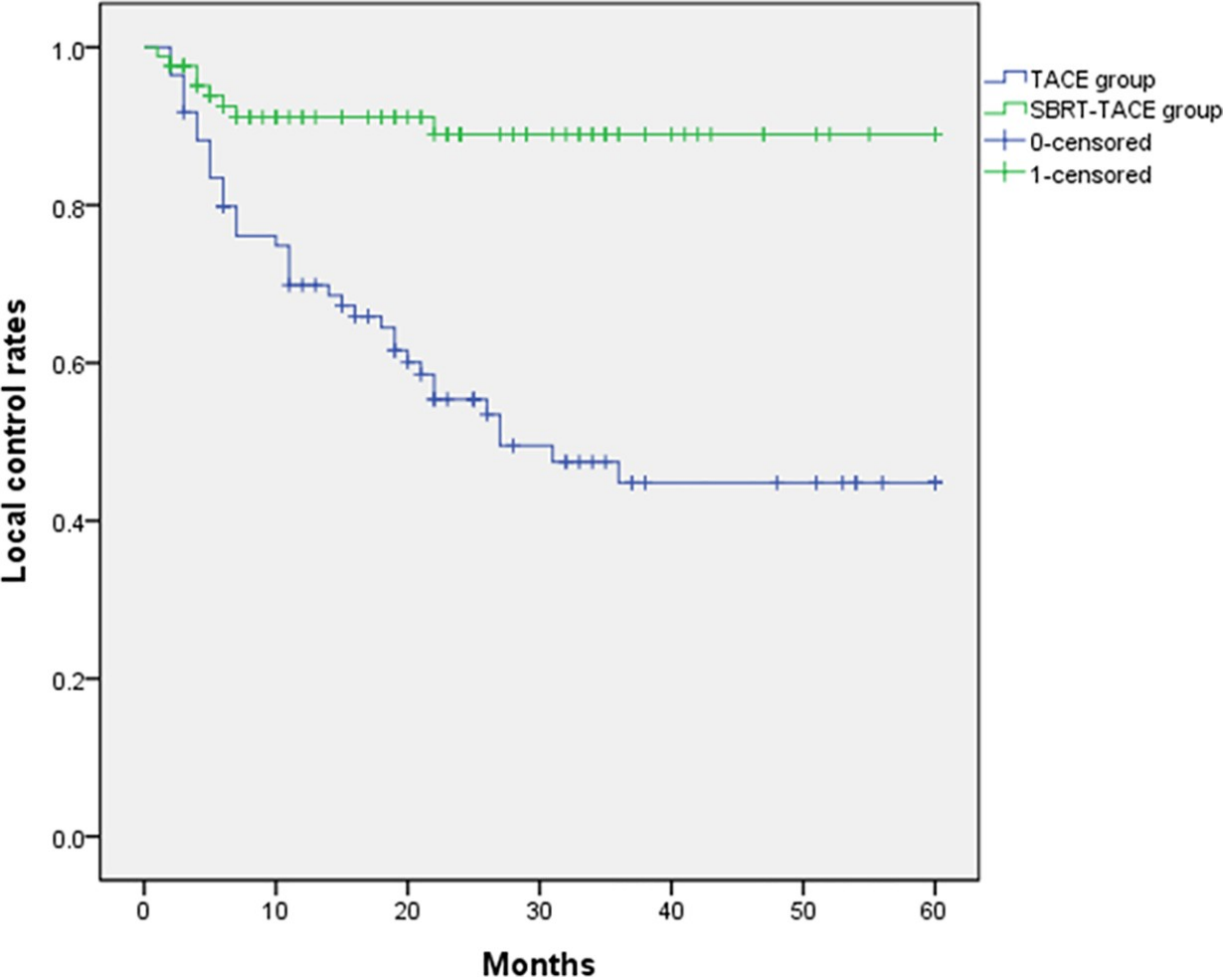
Comparison of the local control rates between SBRT-TACE and TACE groups. (p<0.001).

**Fig 2:**
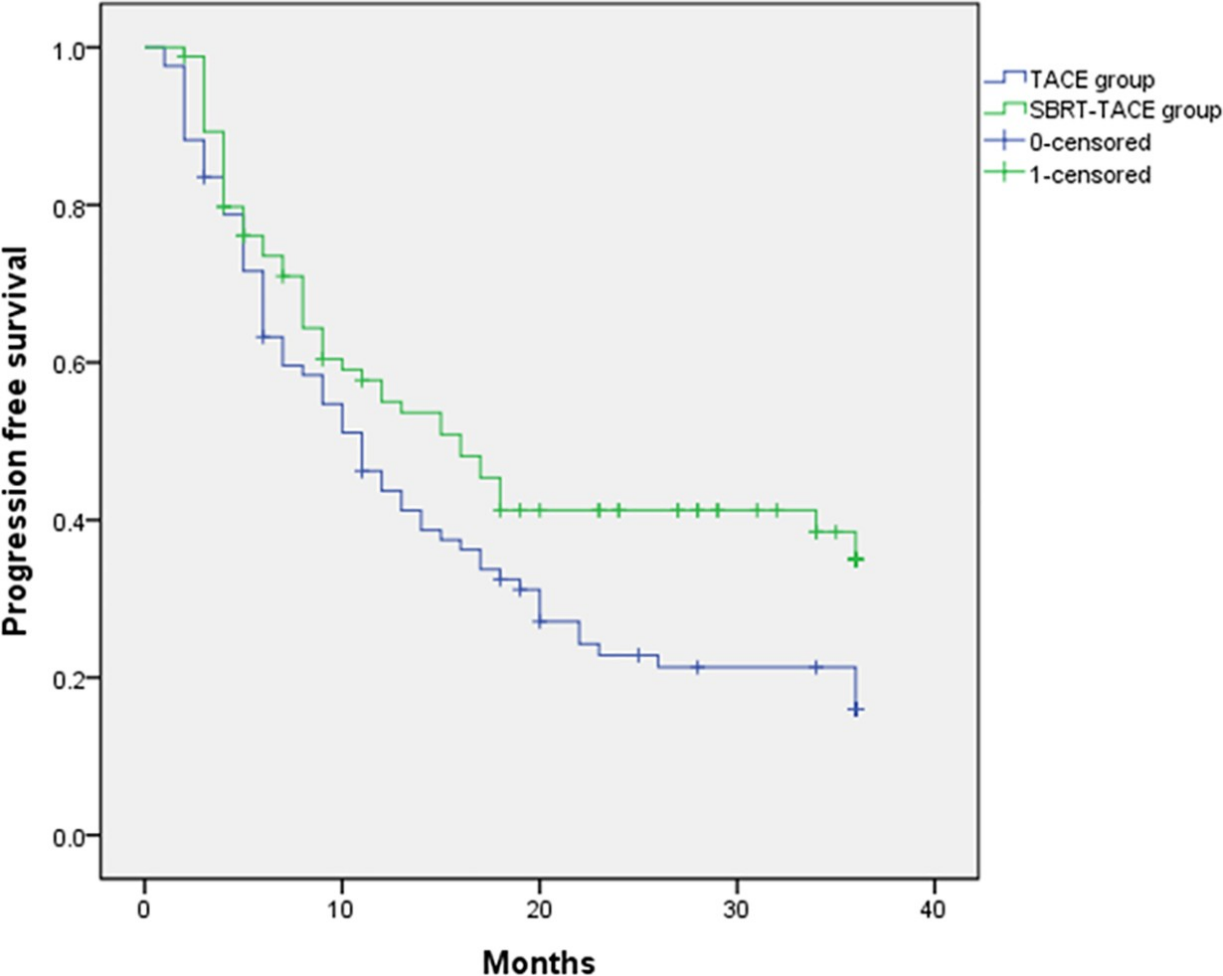
Comparison of progression-free survival rates between SBRT-TACE and TACE groups. (p = 0.022).

**Fig 3:**
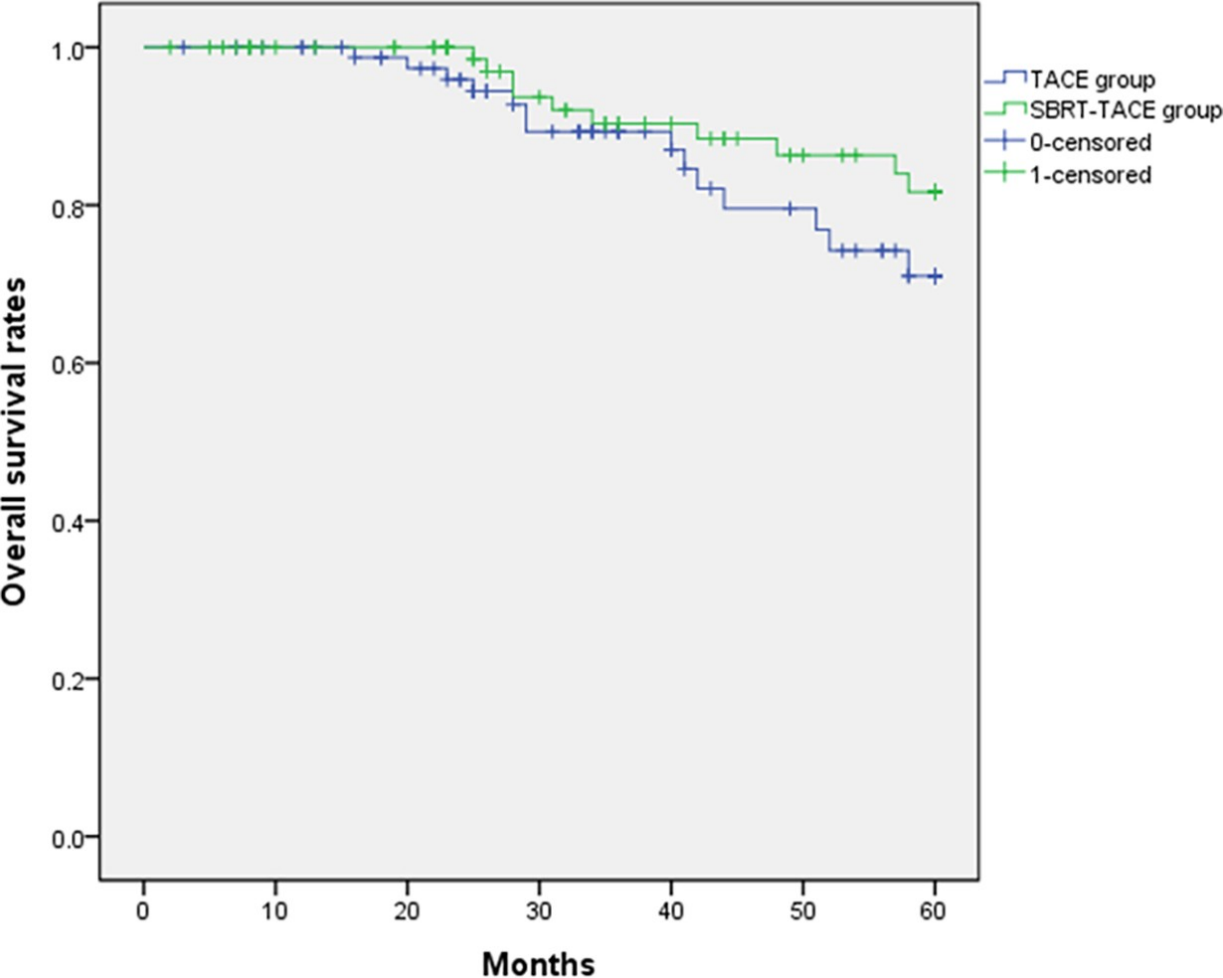
Comparison of overall survival rates between SBRT-TACE and TACE groups. (P = 0.206).

The SBRT-TACE group showed significantly higher 1-, 3- and 5-year LC rates than the TACE group (91.1%, 89.9% and 89.9%, respectively vs. 69.9%, 44.8% and 44.8%, respectively; *P* < 0.001) (Fig 1). The SBRT-TACE group showed better 1- and 3- year PFS than the TACE groups (56.5% and 32.3%, respectively vs. 42.2% and 21.6%, respectively; *P* = 0.022) (Fig 2). However, 1-, 3- and 5-year OS was not different between the SBRT+ TACE and TACE groups (98.8%, 89.1% and 80.7%, respectively vs. 99.7%, 83.3% and 71.0%, respectively; *P* = 0.206) (Fig 3).

### Prognostic factors for progression-free survival and overall survival after propensity score matching

In multivariate analysis, BCLC stage (stage B) (hazard ratio [HR] = 3.701, 95% confidence interval [CI] 1.635–8.379, *P* = 0.002), number of tumors (n≥3) (HR = 2.710, 95% CI 1.494–4.915, *P* < 0.001) and CP class B (vs. A) (HR = 1.945, 95% CI 1.183–3.197, *P* = 0.009) were associated with poor PFS. After adjusting for other variables, SBRT-TACE showed a marginal trend toward significance (HR = 0.695, 95% CI 0.480–1.005, *P* = 0.053) (Table 2).

**Table 2:** Prognostic factors for progression-free survival after propensity score matching.

Variable	Univariate analysis			Multivariate analysis		
	HR	95% CI	P value	HR	95%CI	P value
SBRT-TACE	0.688	0.477–0.992	0.045	0.695	0.480–1.005	0.053
Sex, female	1.009	0.656–1.550	0.968			
Tumor size	1.299	1.107–1.523	<0.001	1.131	0.897–1.426	0.298
Number of tumor			<0.001			0.007
1	1.000			1.000		
2	1.553	1.020–2.365	0.040	1.458	0.910–2.337	0.117
3	3.310	1.986–5.517	<0.001	2.710	1.494–4.915	<0.001
Child-Pugh class						
A	1.000			1.000		
B	2.029	1.242–3.315	0.005	1.945	1.183–3.197	0.009
Age	1.011	0.994–1.029	0.197			
BCLC stage			<0.001			
0	1.000			1.000		0.004
A	1.350	0.885–2.058		1.073	0.665–1.733	0.773
B	6.344	3.170–12.697		3.701	1.635–8.379	0.002
AFP (ng/mL)						
<200	1.000					
≥200	1.130	0.635–2.013	0.677			

HR, hazard ratio; CI, confidence interval; SBRT, stereotactic body radiation therapy; TACE, transarterial chemoembolization; BCLC, Barcelona clinic liver cancer; AFP, a-fetoprotein

CP class B (vs. A) (HR = 2.570, 95% CI 1.241–5.324, *P* = 0.011) and BLCL stage (stage B) (HR = 5.835, 95% CI 1.719–19.801, *P* = 0.05) were significantly poor prognostic factors for OS. Tumor size (HR = 1.179, 95% CI 0.746–1.863, *P* = 0.482) and tumor number (n≤2: HR = 0.582, 95% CI 0.219–1.548, *P* = 0.278) (n≥3: HR = 1.697, 95% CI 0.652–4.417, *P* = 0.279) were not associated with OS (Table 3).

**Table 3:** Prognostic factors for overall survival after propensity score matching.

Variable	Univariate analysis			Multivariate analysis		
	HR	95% CI	P value	HR	95%CI	P value
SBRT-TACE	0.722	0.378–1.380	0.324			
Sex, female	1.021	0.484–2.154	0.957			
Tumor size	1.174	1.018–1.354	0.028	1.179	0.746–1.863	0.482
Number of tumor			0.056			0.303
1	1.000			1.000		
2	0.593	0.228–1.542	0.284	0.582	0.219–1.548	0.278
3	2.426	1.043–5.644	0.040	1.697	0.652–4.417	0.279
Child-Pugh class						
A	1.000			1.000		
B	2.570	1.241–5.324	0.011	2.570	1.241–5.324	0.011
Age	0.989	0.957–1.022	0.508			
BCLC stage			0.068			0.019
0	1.000			1.000		
A	1.755	0.811–3.797		2.231	0.997–4.994	0.051
B	4.367	1.325–14.395		5.835	1.719–19.801	0.005
AFP (ng/mL)						
<200	1.000					
≥200	0.772	0.237–2.511	0.667			

HR, hazard ratio; CI, confidence interval; SBRT, stereotactic body radiation therapy; TACE, transarterial chemoembolization; BCLC, Barcelona clinic liver cancer; AFP, a-fetoprotein

### Subgroup analysis by tumor number

**Fig 4:**
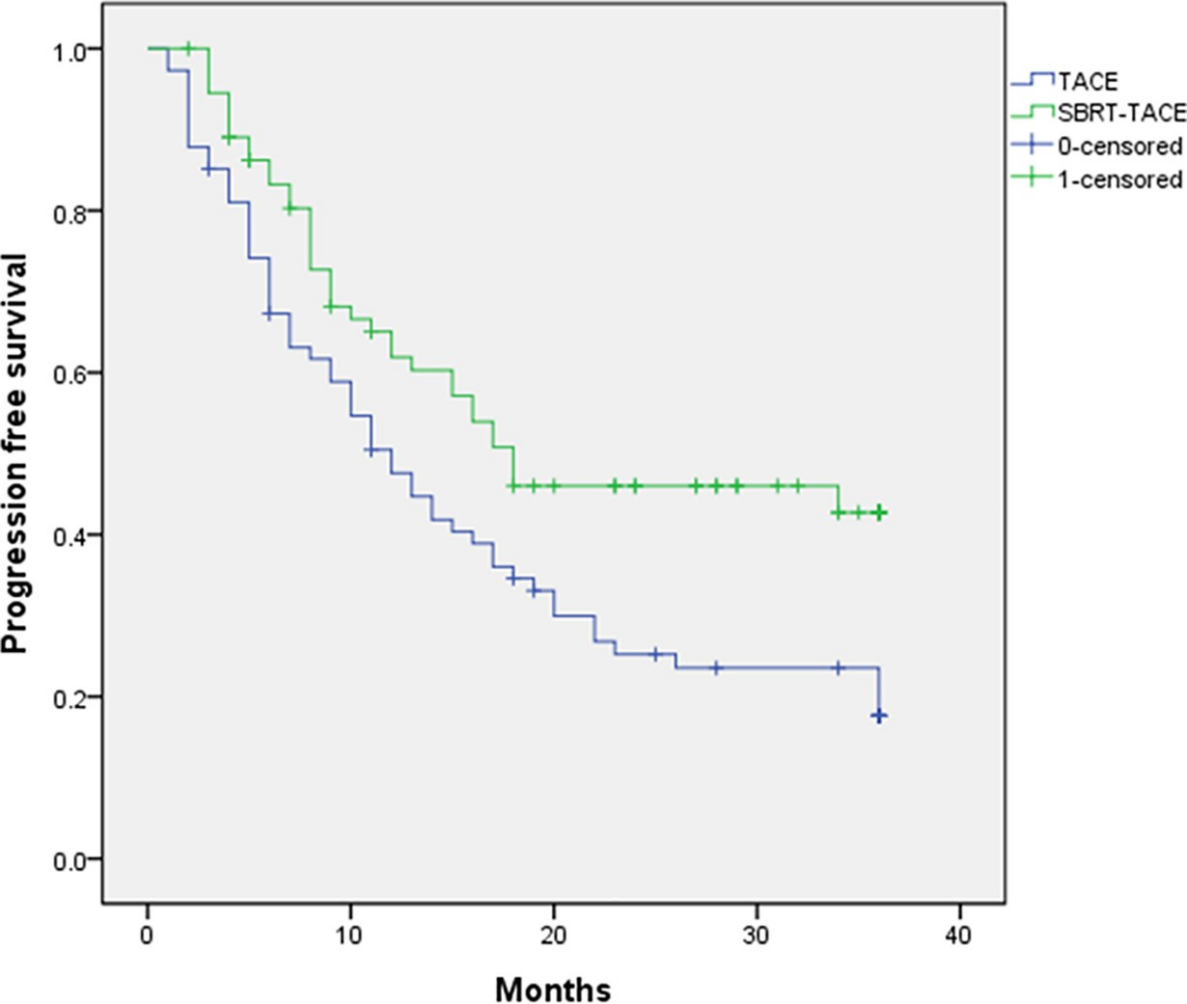
Comparison of progression-free survival with SBRT-TACE and TACE in subgroup analysis by tumor number. (n≤2) (p = 0.006).

Based on previous studies, SBRT was more effective in patients with a small number of HCCs. In our patients with less than two HCCs, the SBRT-TACE group showed better 1- and 3- year PFS than TACE groups (61.0% and 42.2%, respectively vs. 47.6% and 21.6%, respectively; *P* = 0.006) (Fig 4). SBRT-TACE group (HR = 0.590, 95% CI 0.392–0.889, *P* = 0.012) showed a significantly increased PFS after adjustment for BCLC stage tumor size and CP class if a patient had 1 or 2 HCC nodules (Table 4).

**Table 4:** Prognostic factors for progression-free survival in patients with 1 to 2 nodules after propensity score matching.

Variable	Univariate analysis			Multivariate analysis		
	HR	95% CI	P value	HR	95%CI	P value
**SBRT-TACE**	0.604	0.402–0.907	0.015	0.590	0.392–0.889	0.012
**Sex, female**	1.057	0.662–1.668	0.817			
**Tumor size**	1.290	1.141–1.458	0.001	1.132	0.878–1.461	0.339
**Child-Pugh class**						
** A**	1.000			1.000		
** B**	1.925	1.100–3.368	0.022	2.136	1.121–3.762	0.009
Age	1.012	0.993–1.032	0.214			
**BCLC stage**			<0.001			
**0**	1.000			1.000		0.004
**A**	1.186	0.766–1.837		1.226	0.790–1.902	0.363
**B**	5.570	2.363–13.131		6.703	2.817–15.951	<0.001
**AFP (ng/mL)**						
**<200**	1.000					
**≥200**	1.324	0.737–2.376	0.348			

HR, hazard ratio; CI, confidence interval; SBRT, stereotactic body radiation therapy; TACE, transarterial chemoembolization; BCLC, Barcelona clinic liver cancer; AFP, a-fetoprotein

### Liver toxicity

SBRT-TACE and TACE groups showed no difference in liver toxicity after treatment. Worsening of CP score by 2 or more within 3 months after treatment occurred in 8 out of 85 (9.4%) in the SBRT-TACE group and 3 out of 85 (5.5%) in the TACE group, respectively (*P* = 0.119). Elevated liver transaminases more than five times the upper normal limit after treatment occurred in 8 out of 85 (9.4%) in the SBRT-TACE group and 4 out of 85 (4.8%) in the TACE group, respectively (*P* = 0.239).

## Discussion

Only 30–40% of HCC patients undergo curative treatment because many patients with early-stage HCC indicated for resection or local ablation already have advanced liver cirrhosis [19]. SBRT is an emerging technique for patients who are not indicated for radical therapy [20]. In this study, we compared the therapeutic outcome of SBRT-TACE and TACE with propensity score matching. The combination therapy of SBRT and TACE may be more effective than TACE in terms of LC rate. Furthermore, in subgroup analysis, SBRT-TACE in patients with HCCs less than 2 resulted in better PFS without increased liver toxicity. SBRT-TACE represents a favorable alternative for treatment of patients with HCCs less than 2.

As reported in most published clinical studies, SBRT was associated with a favorable LC rate [11, 21, 22]. However, complete tumor response after TACE alone was a challenge [6, 7]. In our study, we reported high LC rate (89.9% at 3 years) in the SBRT-TACE group compared with that of TACE group (44.8% at 3 years). Especially, SBRT resulted in high LC rates in patients with small HCC similar to previous studies. Yoon et al. have reported that LC rate at 3 years was 100% in patients with tumors ≤ 2 cm, and 93.3% in patients with tumors between 2.1–3 cm [11]. Takeda et al also have shown high local control rate (96.3% at 3 years) in HCC (≤ 4cm) patients.[21] In a comparative study of SBRT versus radiofrequency ablation (RFA), similar LC rates were found in patients with small HCC [23]. However, the LC rate of SBRT was not satisfactory with increased tumor size [24, 25].

TACE combined with SBRT has been reported to be effective and safe for the treatment of small- or medium-sized HCC [12, 26, 27]. Jacob et al reported that in patients with HCC tumors measuring ≥3 cm, treatment with TACE-SBRT significantly decreased local recurrence in comparison with TACE alone[26]. In a study from Japan, complete response to therapy was noted in 29 (96.3%) patients belonging to the SBRT-TACE group and in only one (3.3%) patient included in the TACE group (*P* < 0.001) [27]. In a prospective study, SBRT-TACE showed a promising LC rate in HCC (<10 cm) [12]. We also suggest that SBRT-TACE is an effective treatment for both small- and medium-sized HCC with a high LC rate.

In the current study, we achieved better PFS in the SBRT-TACE group than in the TACE group. However, in multivariate analysis, SBRT-TACE was not a predictive factor for PFS. Tumor number (n≥3), CP class B and BLCL stage B were associated with worse PFS (Table 2). According to a previous study, multiple HCC nodules represented an important prognostic factor in PFS [25]. We performed subgroup analysis with HCC number less than 2 and found that SBRT-TACE was a significant prognostic factor of longer PFS (Table 4). In a study, which was limited to patients with HCC carrying 1 to 2 concurrent liver tumors, SBRT resulted in a better PFS than TACE (*P* < 0.001) and TACE was associated with worse PFS in multivariate analysis (HR 3.35, *P* < 0.01) [28].

The effect of SBRT on survival is disputed. A randomized controlled study investigating the efficacy of SBRT and SBRT with TACE has never been conducted. Previous studies reported that OS of SBRT was not inferior to OS following curative treatment. In retrospective studies, OS of SBRT was similar to that of RFA [23, 29]. Su et al suggested that SBRT and liver resection provide similar 5-year OS for small HCC (74.3 vs 69.2%, *P* = 0.405) [30]. However, in this study we did not show that OS of SBRT-TACE was better than that of TACE alone (*P* = 0.206) (Fig 3). Multivariate analysis showed that SBRT-TACE did not increase OS (HR 0.722, *P* = 0.324) (Table 3). Similar to our study, Sapir et al reported no difference in OS between patients treated with TACE or SBRT after propensity score matching [28]. This study did not show difference in OS between the two groups. Possible reasons for such results are described below. First, various treatment modalities were performed for recurrence after TACE only or TACE+SBRT. Second, baseline liver function was similar after propensity score matching. A retrospective study has shown that long-term survival rates after TACE are comparable to those after resection and RFA for small single-nodule HCC. That study explained that degree of baseline liver dysfunction was more important than specific treatment modality itself [31]. Therefore, a prospective controlled trial comparing SBRT-TACE and TACE is warranted to elucidate the survival effect of SBRT-TACE.

We analyzed the differences in liver toxicity after SBRT-TACE or TACE. The incidence of worsening CP score or increased transaminase levels was slightly high in SBRT-TACE group. However, it did not show statistical difference. Many clinical studies have reported that patients did not experience severe radiation-induced liver damage after combined SBRT and TACE [27, 32]. Therefore, SBRT-TACE is a safe option for patients with small HCCs.

This study has a few limitations. First, this study is retrospective. However, with propensity score matching adjusting for potential confounders, this study comparing the benefits of SBRT-TACE with those of TACE was well-balanced. Second, this study is a multicenter study, with variation in devices across multiple institutions. All procedures were performed by the same operator at individual hospitals. However, there was no difference in the treatment methods and response evaluation of HCC between in the four hospitals.

## Conclusion

This study showed that SBRT-TACE compared with TACE is a feasible option for patients with HCC (≤5cm) without increased liver toxicity. SBRT-TACE increased LC rate. We suggest that the advantages of SBRT-TACE should be demonstrated in patients with small HCC. SBRT-TACE is superior to TACE in terms of LCR. Particularly, SBRT-TACE has better PFS than TACE in patients with HCC number (≤2). SBRT-TACE represents an alternative treatment modality.

## Supporting information

S1 Dataset (XLSX)
